# Exploring Professional Support Offered by Midwives during Labour: An Observation and Interview Study

**DOI:** 10.1155/2012/648405

**Published:** 2012-12-04

**Authors:** Stina Thorstensson, Anette Ekström, Ingela Lundgren, Elisabeth Hertfelt Wahn

**Affiliations:** ^1^School of Life Sciences, University of Skövde, P.O. Box 408, 54128 Skövde, Sweden; ^2^School of Health and Medical Sciences, Örebro University, 70182 Örebro, Sweden; ^3^Institute of Health and Care Sciences, The Sahlgrenska Academy, University of Gothenburg, P.O. Box 457, 40530 Gothenburg, Sweden

## Abstract

Support in labour has an impact on the childbirth experience as well as on childbirth outcomes. Both social and professional support is needed. The aim of this study was to explore professional support offered by midwives during labour in relation to the supportive needs of the childbearing woman and her partner. The study used a qualitative, inductive design using triangulation, with observation followed by interviews. Seven midwives were observed when caring for seven women/couples in labour. After the observations, individual interviews with midwives, women, and their partners were conducted. Data were analysed using hermeneutical text interpretation. The results are presented with three themes. (1) *Support as a professional task seems unclear and less well defined than medical controls*. (2) *Midwives and parents express somewhat different supportive ideas about how to create a sense of security*. (3) *Partner and midwife interact in support of the childbearing woman*. The main interpretation shows that midwives' supportive role during labour could be understood as them mainly adopting the “with institution” ideology in contrast to the “with woman” ideology. This may increase the risk of childbearing women and their partners perceiving lack of support during labour. There is a need to increase efficiency by providing support for professionals to adopt the “with woman” ideology.

## 1. Introduction 

Childbearing is a psychosocial event with an impact on the woman and her partner. With their first child, they change from being simply a couple to also being parents [1], and prospective parents describe a need to be better prepared for childbearing [2]. A positive childbirth experience can be of help in mastering this major change in life [3, 4], and support during labour has an impact on the woman's childbirth experience [5]. The childbirth experience is complex, with many related concepts; however, experiencing a sense of control seems to be an important contribution to a positive experience [6, 7]. The childbearing woman needs to surrender herself to the birthing process during labour while retaining an element of control [8, 9].

Professional support from the midwife has been described as providing the childbearing woman with the strength needed to face the challenge of giving birth without losing control [8–10]. When support during labour is continuous, it also reduces the risk of medical interventions, as emergency caesarean section or regional analgesia which are less prevalent, and labours are shorter [5]. 

Support during labour could be offered by the childbearing woman's partner, friend, family members, and professionals [11]. Support is described as an interactive process that is affected by the persons' age, experience, and personality as well as by the environment [12, 13]. Social support from the partner or professional support from the midwife are different; however, both are important for the childbirth experience [14]. In the meta-analysis by Hodnett et al. [5], the effect of support is greater when offered by a person not employed by the hospital and not a member of the social network of the childbearing woman. In recent years, a nonprofessional group called doulas offer continuous labour support to childbearing women and their partners. Research has shown that doulas offer intrapartum support that the midwives seem to be incapable to offer in terms of presence and continuity [15]. Nonsupportive attitudes within organizations and lack of resources such as time or knowledge are described as hindering health-professionals in offering labour support to women [16–19]. The environment, such as conventional or alternative birth setting or leadership, will also affect the efficiency of the support offered and how it is perceived by the childbearing women [13, 20, 21]. Furthermore, women perceiving lack of support during labour may experience intense fear of childbirth in subsequent pregnancies [22].

In Sweden, the midwife assumes responsibility for care offered to women during normal pregnancy, childbirth, and the postpartum period. When complications occur, the midwife will consult an obstetrician who will take over responsibility for the medical care of the woman. During labour, most of the professional support will be the responsibility of the midwife; however, little is known of midwives' perceptions and experience regarding this part of their work. Furthermore, some midwifery students in the USA and Sweden have stated that their tutors did not explicitly address offering continuous labour support [23–25], which made midwifery students feel uncertain of their professional supportive role [25]. Support during labour is important for the childbirth experience and for childbirth outcomes [5], and support could be considered an important part of the midwifery profession [26]. Therefore, the aim of this study was to explore professional support offered by midwives during labour in relation to the supportive needs of the childbearing woman and her partner.

## 2. Method

The present study used an inductive approach with qualitative data collection methods using triangulation [27]. Data were collected through observation [28], which was followed by individual interviews [29] with the midwife, the woman, and her partner within a week of the observation. Data were analyzed using hermeneutical text interpretation [30].

## 3. Setting

This study was conducted in a labour ward in the southwest of Sweden. This labour ward has around 3,000 births per year and serves urban, suburban, and rural areas. Women with both uncomplicated and complicated pregnancies are admitted to the labour ward. The ward is organized with a coordinating midwife, who has the responsibility of handling all incoming communication (i.e., when women in labour call for advice or when they arrive at the labour ward). This organization allows other midwives to care for the woman in their charge without having to attend to answering the phone or dealing with newly-arrived women in labour. The ward has care memoranda stating that the staff should strive to achieve good communication with the childbearing woman to create security and trust, and that the staff should strive to be present with the woman as much as possible. Special attention should be given to first time mothers; however, the midwives have to care for more than one woman simultaneously if the workload is heavy.

### 3.1. The Physical Environment in the Labour Rooms

The labour rooms were quite similar in terms of environment. There was a front room that was delimited from the labour room by a curtain, which could be drawn to block the view from the door if anyone should open it. In the labour room, there was a hospital bed placed in the centre. Along the right side were a wash basin, some stools, and a shelf for weighing and measuring the babies. There were three windows in each room, and curtains varied in colour between the rooms. There were two or three plants on the windowsill. A computer, an electric foetal monitor, a nitrous oxide apparatus, an arm chair, and a bed side table were on the left side of the room. When the observer entered the room, a couple's luggage could have been put on the window sill or placed on the baby cot. Male partners typically wore their own clothes, while mothers more often wore hospital clothes. The midwives wore more or less the same outfit, a blue dress or shirt together with blue trousers.

## 4. Participants

The participants for this study were midwives (*n* = 7) at the labour ward and the childbearing women (*n* = 7) and their partners (*n* = 7) that these midwives cared for during the observation. In order to get a variety of professional and personal experience, the participating midwives were purposely selected [25] out of years in the profession and experience of childbirth. The women and their partners were selected from being cared for by the participating midwives during the observation. All participating midwives were women, and each had from nine to 30 years of practice within labour wards and other areas of midwifery practice. Five of the seven women the midwives cared for in this study were first time mothers and two were having their second child; the childbearing women varied in age from 20 to 37 years and education from secondary school to university education. Their partners were four first time fathers and three were having their second child; they varied in age from 25 to 36 years and education from secondary school to university education. All couples lived together, they were ethnically Swedish, and their living conditions varied from apartments in towns to villas in the countryside.

## 5. Data Collection Procedure

### 5.1. The Observations

Access to perform the study was given by the head of the clinic as well as head of the ward. Midwives that had given their consent to participate in the study were contacted by the first author (S. Thorstensson) to agree on a day for the observation. These participating midwives asked the childbearing woman and her partner for their consent to participate, and a written consent was obtained. The observations started with the first author (S. Thorstensson) entering the labour room and ended after four to five hours or if the baby was born. The observer remained in the room even when the midwife left the room, and careful observation notes were taken continuously. A checklist to capture details and remind the observer of areas of importance was made [26]; however, the observations were otherwise unstructured. The interaction observed included verbal and nonverbal communication, such as eye contact, touch, and where the midwife was focussing her attention [31]. Verbal communication was generally summarised but sometimes noted as quotations The general feeling of the room (as experienced by the observer) and other reflections of the observer (S. Thorstensson) were also noted, as well as time, a description of the room, and the clothes the people were wearing. The observation notes were transcribed within a couple of days from the observation and during this transcription these notes were also completed out of memory and with reflections of the observer specifically noted.

### 5.2. The Interviews

The observation notes formed the basis of the interview with the midwife and with the woman and partner. Consequently, when the observation was completed and before the interviews, the observation notes were read through and notes of interest for the aim of the study were made. The interview with the midwife would also contain general questions about *the responsibility of the midwife in relation to support during labour, and the needs of the woman and her partner.* The interviews with the woman and her partner would also contain overall questions about *their experience of giving birth, being present when the baby was born, and what support needs they themselves or their partners had*. The interview questions were open, and the interviewees were given time to express their thoughts in their own words [29]; when necessary, questions were posed to encourage the interviewees to expand their descriptions. Midwives were interviewed in private within 24 hours after the observation on the labour ward. The midwives were on duty; however, they were released so they could leave for the duration of the interview. Interviewing the women and their partners was done between one and a half and seven days after the birth. The interviews were mostly performed on the maternity ward; however, some interviews were performed in the parents' home for practical reasons. The interviews were digitally recorded and transcribed verbatim.

Observation and interviews were all collected for one observation before the next observation would start. The first author (S. Thorstensson) performed both observations and interviews. Continuous discussions and reflections about data collection and quality of data was done between the first (S. Thorstensson), second (A. Ekström), and fourth (E. H. Wahn) authors, resulting in improvement and amendment some general questions and interview techniques. All observation notes and interviews were transcribed before analysis began. Data was collected from October 2009 to June 2010.

## 6. Data Analysis

Observation note transcripts and interview transcripts were analysed using hermeneutic text interpretation to explore professional support during labour in relation to the needs of the woman and partner. “Hermeneutic” means understanding through interpretation. Interpretation is important for our understanding of the world, and our preunderstanding is necessary for this understanding [30]. The relation between the whole and the parts and the movement in between (the “hermeneutic circle”) is important. Understanding the parts can only be done through understanding the whole, and the whole can only be understood through understanding the parts. Hermeneutic text interpretation is characterized by its focus on the receiver [30]. The interpretation was done in close dialogue with the text and keeping an open mind. Our preunderstanding from working as midwives in labour wards (S. Thorstensson; A. Ekström; I. Lundgren; E. H. Wahn), in antenatal care (S. Thorstensson; E. H. Wahn), and as teachers for midwifery students (S. Thorstensson; A. Ekström; I. Lundgren; E. H. Wahn) could assist when interpreting the text. However, our preunderstanding could also obscure the meaning of the text and hinder in seeing something new. To keep a dialogue and ask questions to the text, keeping an open mind, and continuously reflect about our preunderstanding, assisted when interpreting the text [30]. One way to identify whether the preunderstanding is dominating the result is to look for parts in the data and in the result that are conspicuous or surprising [32].

The interpretation started by reading the observation notes and interviews with an open mind to gain a first general understanding of professional support by midwives' during labour within each observation and linking this with the interviews with the midwife, the woman, and her partner. Then, the texts were read through again, and notes were made and questions arising were noted. Answers to the questions were then looked for in the data. The analysis continued interpreting the text in parts by identifying strophes of the same meaning and getting an idea of the horizon of the text and the horizon of the interpreter [30]. The questions asked to the text were as follows: *How can we understand midwifery professional support during labour*? *How will midwifery professional support present itself during labour? How will the woman and/or her partner experience these situations?,* and *What supportive needs does the woman or her partner express during labour?* The findings were organized in themes. Then, the themes and the text were read through to search for a new whole (a main interpretation), moving from the whole to the parts and back to the whole again [33]. The main interpretation was structured at a more abstract level and the concepts “with woman” and “with institution”, as described by Hunter [18], were used in order to explore professional support during labour in relation to the needs of the woman and her partner.

## 7. Ethical Considerations

Before data collection began, information about the study was sent to antenatal clinics to inform parents about the study so they would be prepared if they would be asked to participate. Observing during labour is a delicate situation, and there is a risk that the woman, her partner, or the midwife could experience the observer as an intruder. Both the midwife and the couple were informed that they could ask the observer to leave at any time had her presence been disturbing to them. There could also be situations arising during the observations that demand action from the observer from an ethical standpoint, and the observer (S. Thorstensson) had mentally prepared for that. However during the observations in this study, no such situation occurred. Ethical approval for the study was obtained from the Regional Ethical Review board in Gothenburg before data collection began. The study was conducted in accordance with the Declaration of Helsinki (1964).

## 8. Findings

The findings are presented in three themes*. Support as a professional task seems unclear and less well defined than medical controls; midwives and parents express somewhat different supportive ideas about how to create a sense of security; partner and midwife interact in support of the woman.* The main interpretation is presented at the end of the present section.

### 8.1. Support as a Professional Task Seems Unclear and Less Well Defined than Medical Controls

The midwives attention to supportive needs varied and seemed to be based in their own idea of the purpose of care during labour and on their own personal experience of childbirth. Further, the midwives paid obvious attention toward medical control of the physiological process (i.e., planning and informing when the next vaginal examination would be performed). When in the room, the midwife would give the woman her full attention; however, the midwife's reason for entering the room seemed primarily to be in order to perform tasks such as medical controls.

*The midwife sits beside the woman with her fingers on the woman's belly. The contraction is over, the midwife checks the blood pressure and it is now somewhat lower than before. The midwife says that this tells her that the epidural has started to take effect. The midwife takes the trolley for epidural anaesthesia out through the door, but first she puts a blanket over the woman. The midwife says to the woman, “I will send in the auxiliary nurse to check your blood pressure”. (Observation notes, couple, second child).*

The midwife's main focus of attention seemed to be on control of the physical process and offering support in between. However, the midwife would also sometimes focus on offering support to the woman and her partner in handling the labour process, and then her attention at performing tasks such as medical controls would be placed more in between. While medical controls seemed to be planned and performed with consistency, supportive needs sometimes seemed to be addressed with uncertainty or not at all. This uncertainty could emerge from the idea described by the midwives that there is a right (and, consequently, a wrong) way to do things in relation to supportive needs. This idea seem to stem from a lack of knowledge in meeting supportive needs, which leaves the midwives with only their own experience (i.e., their own childbirth experiences) when offering support to the woman or her partner.

*Contraction—the woman breathes nitrous oxide. The midwife turns to the computer and writes. The woman puts her hand over her lower back. She let's go of the nitrous oxide and takes a deep breath. (Observation notes, woman, first child).*

*Midwife; Well, it is difficult to … how can one know how … // … I could imagine that most perhaps want to be left alone … // … yes, I do not want other people to fuss with me when I have a contraction … (Interview, midwife).*

Different needs of the woman and the partner were observed during labour. The need for energy or to visit the toilet seems clear, unambiguous, and well defined. These needs were actively met by the midwives during labour. However, some needs, such as the woman's need for reassurance or the woman (or her partner) being worried, seem ambiguous, vaguely expressed, and less well defined. These needs sometimes seemed difficult for the midwives to understand; consequently, they were not always met.

*The woman says, “how bad will it hurt?” The midwife does not answer or react to her question. (Observation notes, woman, first child).*

Meeting needs of reassurance seems to demand that the midwife interprets what the woman's actual need was, rather than offering direct action or answers. When observed to address concerns or provide reassurance, the midwives used information or general terms of reassurance; however, they sometimes seemed uncertain or hesitant. Nevertheless, when midwives in the interviews described clear ideas of supportive care in labour; this appeared to have helped—for example, in meeting insecurity by offering reassurance—as the following excerpts illustrate.

*The woman has a contraction. The midwife sits with the couple, the partner is holding the woman. … // … Another contraction. The woman is groaning, and her partner is holding her. The midwife is holding the woman's knee. (Observation notes, couple, first child).*

*Midwife; Every time she had a contraction, I held on her knee or something only to make her feel that, even if it is painful for her … she can feel that I am calm and sort of think that this is ok … I try to communicate that … (Interview, midwife).*

*Woman: … when she had her hand on my knee, that I remember because it felt really good (“good” stated with emphasis) it was during the contractions that she held her hand on my knee and it felt really good … but the belly I did not notice; I guess it was that she wanted to feel the contraction … but when she had her hand on my knee … that felt good (“good” stated with emphasis) … (Interview, woman first child).*

The midwife describes a clear intention to communicate reassurance to the woman through touching her knee. The intention the midwife describes for her nonverbal action seems to influence the woman's experience of this action accordingly.

Where the midwives place their attention seems to affect whether they meet only clearly expressed needs, or also needs that were ambiguous and vaguely expressed. There seemed to be more consistency in performing medical controls than in offering support to the woman and her partner. This difference seems attributable to the midwives varying in certainty or knowledge when addressing supportive needs.

### 8.2. Midwives and Parents Express Somewhat Different Supportive Ideas about How to Create a Sense of Security

To support a sense of security for the women and their partners, the midwives offered information continuously during labour. This information was mainly practical (e.g., if leaving, when the midwife would return) or medical (e.g., how an epidural works). The midwives described in the interviews that credible information was crucial for the parents to trust the midwives. Women and their partners stated that continuous information created a sense of hope and security.

*Interviewer: … could you give me some example of something that the midwife did that made you feel secure?*

*Woman; no … well … yes … they said what they were going to do … // … and that sort of she said that she would come back, and when she said she would come back she did that also … (Interview, woman, first child).*

When the midwives informed parents about procedures not directly in connection with labour, or gave information that was contradictory to what the woman or her partner believed to be true, this information was not considered supportive. Information was also described by the midwives as a way to prepare for medical intervention, though with a need to balance when and how much information should be offered, since information can be calming but can also be worrying. Information could also be indirect, such as offering spinal anaesthesia that would only last three hours, indicating to the woman/couple that the baby would be born within this time, even if the midwife did not say this explicitly.

One aspect of support to create security is to invite the woman to participate through awaiting her readiness before performing examinations and this was done by all midwives. The midwives also invited the women and their partners to participate in decisions. However, this invitation sometimes seemed somewhat dubious, since the woman, for example, could find it quite hard to actually say no to the midwife leaving the room. Even if the midwife technically presented her proposal as a suggestion, it seemed more as information and not as an invitation to participate in the decision.

*The midwife asks the couple, “is it ok if I go in and out of the room a little?”*

*(Observation notes, couple, first child).*

* Midwife: well … what I think about then is that they in some way should get a chance to be involved in the decision in some way … that I do not just leave and they do not know how I had thought … (Interview, midwife).*

The midwives' presence in the room seemed to be supportive in creating a sense of security. For the woman to be able to look up and meet the eyes of the midwife could be reassuring. As a consequence, when the midwife leave the room, she would take away some of this sense of security. When the woman was very secure with her partner, she described not being so aware of the midwife leaving the room. However, the partner described a sense of responsibility when left alone with the woman during labour, and this seemed to have created insecurity for the partner.

*Partner: They told you exactly what to do, and they were very helpful the whole time, so it felt good when they were present. It was worse when they left sometimes, they ran in and out in between, and that (made me) nervous (he laughs) … // … well, then (I) felt a bit nervous, then I thought, “well then, I am on my own, then” … (Interview, partner, first child).*

The midwife could also be more continuously present in the room and wait for the contractions alongside the couple. In this case, her absence would not create insecurity in the same way and her absence was described as if she was still in the room.

*Partner; well … she went out and then it was just to continue as if she was still there … (Interview, partner, first child).*

The midwives did not explicitly describe their own presence in the room as important, even if they mention that offering time is important to support creating security during labour. Even when the woman has an urge to push and she wants the midwife to stay in the room, the midwife could chose to leave if another task was deemed as important and the midwife felt certain that birth was not imminent.

*Woman: … well it was that last period when I started to feel the urge to push, and she had this other patient to report, and so … so then I got a bit frustrated that she disappeared; I did because then I felt that now the baby is on the way and then it was still two more hours (she laughs) really … but … it did feel a bit … then it was a bit scary that she left actually … (Interview, woman, first child).*

*Midwife: Eh … I knew she had good support from her husband and that she would not give birth right now and I had this other woman that I had to attend to … // … *

*Interviewer: How do you think she experienced this that you left there?*

*Midwife: She and many with her will first feel some panic because of their urge to push, and they do not want to be alone—but if I explain again so that she can understand … // … it was the same with her or with them, they understood, and she did not ring the bell, but she was secure in this … (Interview, midwife).*

When the midwife can joke or make small talk, the couple seems to perceive that the midwife is in control of the situation, and this supports a sense of security for the woman and her partner. Women described how being in labour made them feel different and that this could be frightening. If the midwife then made small talk, this created a sense of the usual, and the woman regained a sense of her usual self. The midwives were observed using jokes and small talk in interacting with the women and their partners. When interviewed, the midwives said that this was because *it is so nice to small talk a little or I am a joking kind of person*. The midwives seemed a bit uncertain as to whether joking is appropriate in the situation; they do not describe it explicitly as a supportive strategy.

There seemed to be somewhat different ideas of support to create a sense of security. The midwives and the women/couples agreed on continuous credible information being important when supporting to create a sense of security. That the midwives presence in the room or making small talk and joking would support to create a sense of security did not seem to be entirely acknowledged by the midwives. However, to the woman and her partner, the presence (or absence) of the midwife, as well as joking, and small talk, seemed important to their sense of security.

### 8.3. Partner and Midwife Interact in Support of the Woman

The midwives and partners were observed to interact when offering support to the woman. This interaction could look almost like a “dance”, where the midwife would suggest something and the partner would act accordingly in support of the woman, or the partner would act and the midwife would confirm his action.

*The midwife says to the woman, “it seems as if you can easier handle your contractions when you are standing” … // … (Another contraction). The woman breathes through the contraction, she is grimacing and she holds the partner's hand. The contraction is over. He says immediately: “ok, now you get up”. The midwife also says, “it is good to be upright and walk, the second best is to sit in the armchair”. (Observation notes, couple, second child).*

If the partner is a first time parent, the midwife may need to offer more guidance on how to support the woman than if he was expecting his second child. The midwife then invited the partner by asking him what he thought about his role during labour. When invited by the midwife, the partner described feeling involved in the process. Interaction between midwife and partner in support of the woman seems difficult when the partner and the woman do not agree. One example of this is if the woman feels she is handling the contractions, but her partner has difficulty in handling her having pain.

*She is breathing and groaning. He looks at her and says, “what do you think, should you try nitrous oxide?” She answers, “I feel rather ok”. He replies, “You do not look ok to me” … // … He asks the midwife, “is it time to think about anaesthesia now?” The midwife replies, “yes if you (she turns to the woman) feel that you need something more, then it is time for that now” … (Observation notes, couple, first child).*

This supportive situation calls for a delicate “act of balance” for the midwife, in differentiating between the actual needs of the woman and the demands or needs of her partner. The midwife may also interact with the partner in support of the woman, as well as the physical process. Helping the woman to relax could be one way of doing this.

*The midwife says, “Do you want to lie down?” (Another contraction) The woman says, “ouch … ouch” She breathes through the contraction. Then she lies down in bed. The midwife says to her partner, “You can lay down behind her if we pull up the “gate” so you do not fall out of the bed”. // … He lies down behind her in bed and holds her. They talk between contractions … // … The midwife asks, “is the pain most in front?” The woman is nodding. The midwife places a warm cloth over the woman's symphysis and belly and then pulls the blanket over them both. The midwife looks at them and says, “this looks quite cosy”. (Observation notes, couple, first child).*

The woman could be very tired and vague about her needs; in these cases, the midwife could interact with the partner to reach a contact with the woman. The partner could ask the midwife questions on behalf of the woman. However, it was also observed that sometimes the midwife did not interact with the partner in support of the woman. The midwife could enter the room with her attention toward the woman and tasks needing to be performed. Then the midwife could place herself between the woman and her partner as if he was not present. This could seem to make the partner feel uncomfortable, as if he was in the way and did not have a place or function in the room. However, if the partner feels that the most important thing is that the midwife takes good care of his wife, then he may not experience this as awkward. This situation may be easier to handle for a partner expecting his second child.

The midwives would address the partners need for drink or food in various ways. However, when asked about the need of the partner, it varied as to whether the midwives did reflect about the partner and what needs he might have. The needs mentioned were the need for participation and information. However, one midwife concluded that “*he has the same need as her … it is just that he is not in pain …”*.

The midwives' seemed to vary in interacting, meeting, and supporting the partners' need to act supportive towards the woman. There seemed to be a continuum where the partner could be seen, on one hand, as someone who is not really involved in the situation, or, on the other, the partner could be seen as important, involved, and in need of guidance and support to fulfil his part.

## 9. Main Interpretation

Midwives' supportive role during labour could be understood as being mainly affected by the midwife adopting the “with institution” ideology in contrast to the “with woman” ideology, as described by Hunter [18]. These ideologies should not be seen as stressing or neglecting medical safety but merely as an illustration of differing perspectives in relation to professional support. In the “with institution” ideology, professional support is unclear, vague, and based on the midwives' personal experiences. In the “with woman” ideology, support is clearly offered within the professional role and in cooperation with the partner. When the midwives place their attention only at tasks, with a consistency in performing medical controls, it could be understood as the midwives adopting the “with institution” ideology with an attention to efficiency, only focusing on physical safety and risk-management. Supportive needs that are more ambiguous or vaguely expressed could then be understood as unimportant for the midwife, which could lead to clearly expressed needs, such as the need for food or drink, being actively met, while ambiguous needs, such as the need for reassurance, being addressed with uncertainty or not at all. This uncertainty could be reinforced by the idea described by the midwives that there is a right and, consequently, a wrong way to do things in relation to supportive needs. Uncertainty in addressing ambiguous supportive needs could be understood as the midwives lacking knowledge about how to address supportive needs.

On the other hand, when the midwives adopt the “with woman” ideology, their focus would be to form and sustain a relationship with the woman/couple. To focus a relationship could be understood as making the midwife more aware of and prepared to meet both clear and ambiguous supportive needs of the woman or her partner. The somewhat different ideas about how to support to create security during labour could be understood as a contradiction between the woman and her partner expressing a wider range of supportive needs to experience a sense of security in labour, while the midwives adopting the “with institution” ideology would mainly focus on information and tasks to perform. Being present in the room with no actual task to perform apart from offering support, or to joke or make small talk, could be understood as inefficient under the “with institution” ideology. However, when adopting the “with woman” ideology it would be efficient for the midwife to be present and to joke and make small talk in order to form and sustain a relationship with the woman and her partner. This could be understood as the “with woman” ideology being more adequate in meeting the wider range of supportive needs described by the woman and her partner, than the “with institution” ideology. In the “with institution” ideology, the woman's partner is present as support to her, and their becoming parents is not acknowledged as important, since emphasis is only on task performance and risk-management. However, when adopting the “with woman” ideology, support from the midwife could be understood as addressing both the woman and her partner as becoming parents and to interact with the partner in support of the woman during the birth process.

## 10. Discussion

The most important finding in this study was that professional support, offered by midwives during labour, is affected by the ideology adopted by the midwives. The adopted ideology will affect which supportive needs of the woman/couple the midwife will address during labour.

Midwives, women, and their partners describe somewhat different supportive ideas about how to create a sense of security during labour. The midwives, when adopting the “with institution” ideology, will only focus on efficiency and mainly offer information though women and their partners express a wider range of supportive needs to experience a sense of security during labour. Labour is a complex, changeable, and flexible process [34], and the physiological process can be disturbed if the woman does not feel secure enough during labour [35, 36]. To feel secure during labour, the woman needs presence of supportive persons that can offer security in the process [9, 37, 38]. Offering support during labour needs to be almost continuous in order to be effective, which could, theoretically, be understood as decreasing stress reactions and imposing security in the labouring woman [5]. Findings of this study suggest that supportive actions were less well defined as professional tasks than task, such as medical controls, and the midwives, relying mainly on personal experience, seemed uncertain in meeting ambiguous supportive needs. This uncertainty could be understood as the “with institution” ideology imposing knowledge of supportive actions as not important. Knowledge could be silenced or not acknowledged as important (i.e., tacit knowledge) as a result of power or status imbalance [39], which could lead to midwives adopting the ideology “with institution”, perceiving lack of knowledge to offer support. Lack of knowledge in offering support during labour has been described elsewhere [16]. Thus, it may be due to the domination of the ideology “with institution” that supportive actions as forming and sustaining relationships are important, but hidden, aspects of care in labour [40].

Findings of this study suggest that midwives vary in how they interact with the partner in support of the woman during labour, which could be understood as the midwives adopting different ideologies. The partner most often wants to offer support to the woman during labour [41–43], and, depending on which ideology the midwife adopted, they may overlook “with institution” or actively meet “with woman” the partners' need for guidance and support. Findings from this study described that the partner expressed uncertainty when being left alone to support the woman during labour, while the midwives did not entirely acknowledge their presence as important. This is in line with earlier studies in which partners report feelings of helplessness and anxiety during labour [44, 45]. These feelings of anxiety could impose feelings of being left out if the father was not supported [42], and professionals being present was described by the partner as important support [46].

Findings from this study also suggest that it could be complicated for the partner to handle his own reactions to the woman's pain, as well as supporting her during labour. These results emphasise that the partner needs guidance to fulfill his supportive part [41–43, 47, 48]. Hence, when midwives adopt the ideology “with institution”, with emphasis only on task performance and risk management, they could be less efficient in meeting supportive needs of the partner than when midwives adopt the “with woman” ideology.

The “with institution” ideology only focusing on efficiency could be understood as prompting that midwives should be able to attend to more than one woman in labour when necessary though actually attending the one woman in labour when possible. However, the main interpretation of this study suggests that the differing ideologies propose different focuses, such as a focus to meet supportive needs of the woman or her partner, or a focus on task performance. Hence, it might be difficult for midwives to alternate between the differing ideologies in response to the workload of the ward. On the other hand, continuous labour support has been shown to be efficient in reducing medical interventions and to increase the chance of a positive birth experience. However, this support seems more efficient when offered by nonprofessionals [5]. Perhaps professionals more often adopt the “with institution” ideology, as the midwives in this study mainly did, which may lead to professionals failing to offer efficient labour support as result of this study suggest.

Women describe the experience of midwives as uncaring during labour [49], or women experience lack support from the midwife during labour, which sometimes results in intense fear of childbirth in a subsequent pregnancy [22]. In relation to the findings of this study, it could be suggested that, when midwives adopt the “with institution” ideology, their ability to meet supportive needs decreases. That will increase the risk of women experiencing lack of support during labour. Support in labour has an impact on the childbirth experience [5], and six percent of women that reported dissatisfaction with care during labour were unsatisfied with interpersonal care [50]. Perhaps organizing for professionals to be able to adopt the “with woman” ideology would be more efficient when both medical and care outcomes are concerned, since this could lead to less women experiencing lack of support during labour. However, adopting the “with woman” ideology seems to present midwives with more emotional labour [18] in relation to colleagues and the organization than when adopting the “with institution” ideology, suggesting that support within the organization is essential for midwives to adopt the “with woman” ideology.

## 11. Method Discussion

The strengths of this study include the method used, which offers an opportunity to enter deeply into the supportive role of midwives. The triangulation in data collection [25] provided both observed data and the thoughts and reflections of both midwives and the women/couple that they cared for. However, as with all qualitative research, the results must be interpreted in relation to the context [51]: in this study, a single labour ward in Sweden. On the other hand, both the midwives and the couples they cared for during the observations varied in experience, parity, age, and education which could strengthen the trustworthiness of the result [25]. The fact that the results are contextual does not mean that it has no meaning in other contexts; however, they must be related to the new context, which is in line with the hermeneutic circle [51].

It is not possible to perform an observation study without having an impact on the situation under study. However, the observer tried to move as little as possible in order not to disturb the midwife or the couple. Neither the midwives nor the couples felt disturbed by the observer when questioned about it in the interviews. When using hermeneutic text interpretation in analysing data, our preunderstanding could assist as well as hamper us when exploring [30] midwives' supportive role during labour. Analysis was done with an open mind and complemented by constantly challenging our preunderstanding through reflection [52].

## 12. Conclusion 

Midwives' supportive role during labour was affected by them mainly adopting the “with institution” ideology rather than the “with woman” ideology. Adopting the “with institution” ideology, the midwives place their attention mainly on task performance and risk-management, which increased the risk that supportive needs of women or their partners during labour would not be met. When adopting the “with woman” ideology, it seems that a wider range of supportive needs expressed by the woman and her partner will be met by the midwife. However, to meet the sometimes-contradictory needs of the woman/couple and the requirements of the institution might give rise to emotional strain for the midwife. Hence, the organization in which the events take place needs to increase efficiency by providing support for professionals to adopt the “with woman” ideology to increase the chance of the supportive needs of women and their partners being met.
